# Progress of Surgery

**Published:** 1875-03

**Authors:** Jno. E. Owens

**Affiliations:** Lecturer on Surgery, Rush Medical College, Chicago


ARTICLE III.

### PROGRESS OF SURGERY.

By JNO. E. OWENS, M.D.,

LECTURER ON SURGERY, RUSH MEDICAL COLLEGE, CHICAGO.

1. Practical Indications to be Drawn from the State of the Pupil during Surgical Anæsthesia. By M. P. BUDIN. (*Le Progres Medicale*, Sept. 5, 1874; *The American Journal of the Medical Sciences*, January, 1875.

2. New Method of Operating on the Larynx. By Dr. A. EYSELL, of Halle. (*Med. Times and Gazette*, Oct. 17, 1874; *The American Journal of the Med. Sciences*, January, 1875.)

3. Extirpation of the Entire Larynx. (*British Med. Journal*, Nov. 28, 1874; *The American Journal of the Med. Sciences*, January, 1875.)

4. Luxation of the Penis. Dr. MOLDENHAUER. (*Berliner Klin Wochenschrift*, No. 45, 1874; *Phil. Times*, Jan. 2, 1875; *The Clinic*, Jan. 9, 1875.)

5. Influence of Oxygen upon the Formation of Pus. By Prof. BINZ. (*Schmidt's Jahrbucher*, Oct. 28, 1874; *The Clinic*, Jan. 9, 1875.)

6. Resection of the Hip in a Child of Twenty-one Months. By Dr. SAYRE. (*N. Y. Med. Journal*, December, 1874; *The Med. News and Library*, January, 1875.)

7. Bacteria. By M. SERVEL. (*Gazette des Hopitaux*, Dec. 8, 1874; *The Clinic*, Jan. 16, 1875.)

1. In his endeavor to discover some sign which might serve as a guide in the administration of chloroform, and which would indicate the state of sensibility during its administration, Dr. Budin has arrived at the following conclusions :

*a.* There exists in surgical anæsthesia produced by chloroform a constant relation between the state of the pupil and the period of anæsthesia.

*b.* During the period of excitement the pupil is *dilated.*

*c.* This period having passed, the pupil contracts; this atresia is well marked, and continues for several minutes, accompanied with complete general anæsthesia.

*d.* The *dilatation* of the pupil coming on during an operation indicates, in general, either that the anæsthesia is less profound, or that the return of sensibility is approaching.

*e.* The state of the pupil, then, may serve as a guide in the administration of chloroform.

*f.* During operations which last a long time, if it be wished that the patient should be insensible and motionless, the anæsthesia should be so managed that the pupils continue constantly contracted.

*g.* Finally, *vomiting* may produce dilatation of the pupils, cause the insensibility to disappear, and the patient to awake; it annuls, in part, the effects of the anæsthesia.

2. An account of a new method of conducting operations on the larynx appeared in the *Centrablatt fur Chirurgerie*, for Aug. 15th. Every one, says the author, who employs the laryngoscope must be aware how difficult it is to reach a tumor growing in the lower part of the larynx, which is not sufficiently movable to be driven above the level of the vocal cords by forced expiration. The following is Dr. Eysell's method: Whilst observing the larynx by means of the laryngoscope, for which he employs either daylight or the electric light, an exceedingly elastic needle is passed through the skin and cricothyroid membrane, into the larynx, exactly in the middle line, and immediately beneath the thyroid cartilage. The needle is then made to transfix the tumor, and by depressing its handle the latter is forced up into the ventricle of the larynx. No local mischief has followed even frequently repeated operations. If it be intended to cauterize or tear away the tumor, the patient is directed to hold either the mirror, or, better still, the needle. In this way Dr. Eysell has succeeded in removing two fibromas from the lower part of the laryngeal cavity. The needle is rendered more pliable by subjecting it to heat.

In a case where the vocal cords were adherent to one another for their anterior two-thirds, the result of a suicidal cut throat, which caused considerable shortness of breath on slight exertion, a narrow tenotome (0.5 centimetre broad) was passed through the scar into the larynx. When the point appeared behind the triangular adhesion, the handle was firmly depressed, and by drawing the knife downwards the cords were separated almost to their origin.

3. Prof. Billroth, of Vienna, performed the operation for the second time on Nov. 11th. The patient, a man aged fifty, suffered from epithelioma, which made rapid progress, the whole of the interior of the larynx becoming affected, and the dyspnœa constantly increasing. A favorable prognosis was given, with regard to the return of the disease, on the ground that no infiltration of the neighboring lymphatic glands could be detected. The patient died on the night of the 16th, apparently from pneumonia.

4. In the case described, the penis was torn completely from its enveloping skin, thrust beneath the coverings of the abdomen and there imbedded in the fat and connective tissue. The organ was discovered by incision in its abnormal position eighteen days after the occurrence of the accident. The urethra was said to be uninjured ; the penis had formed adhesions to the abdominal muscles ; a catheter could be passed from the meatus to the symphysis, where its further progress was arrested. The glands and prepuce were dissected off, but, at the request of the patient, who stated that he only wished the penis for the function of urination, no further attempt was made to restore the organ to its normal condition. That which had been regarded at the time of the injury as the crushed and bruised penis was composed only of the cutaneous envelop of that organ, the deeper portions of which had been dislocated as described. The wound finally healed, the penis up to the glans being adherent to the muscular structure of the part and covered with skin. The glans having been left bare and movable, urination was accomplished without trouble. The patient was a strongly-built farmer, aged fifty-seven. Having fallen from his wagon, the horse forced him against a tree, and the wheel of the wagon passed close in front of his body, causing the injury above described.

5. Prof. Binz considers the migration of white blood corpuscles as closely connected with the presence of oxygen. During the repetition of Cohnheim's experiments with the mesentery of hogs, he observed that at the time of the formation of pus, the smallest vessels are only filled with plasma and white corpuscles, which are subject to at least as great blood-pressure as in the neighboring vessels; as soon as the red corpuscles find their way here, the well-known movements begin. As the white cells are either formed anew by the irritation of the wound, or are forced into the circulation and seen to cling to the walls, they receive the first stimulus of the oxygen brought by the red corpuscles, and commence the amoeboid movements.

6. The child had been attacked with double pneumonia, and, shortly after recovering from it, fell on its left side, with the limb abducted. Some weeks later, an inflamed gland was discovered in the inguinal region, which was followed by an abscess. The abscess was opened and allowed to discharge. The limb was œdematous when seen by Dr. Sayre, who extended the incision and found erosion of the end of the femur. At the end of a week the head of the femur was removed, the anterior opened, closed, and a more dependent one substituted. The little patient did well, though it had never received any nourishment except from its mother's breast.

7. M. Balard presented to the Academie des Sciences, Paris, in the name of M. Servel, a communication on the generation and evolution of bacteria in organic tissues protected from contact with the air. The author was induced to make his experiments after having noticed that solutions of chromic acid hardened only the outside of thick anatomical specimens, without preserving the central portions from decomposition.

Thus prompted, M. Servel prepared a one per cent. solution of chromic acid, into which he plunged two living tetes de cobayes which he had just decapitated. After six days of immersion the external coverings of the head were preserved ; but the brain and central portions were evidently in a state of corruption. Examined with the microscope, the cerebral pulp presented a great number of bacteria of all sizes.

These experiments might furnish subject for criticism on account of the possible presence of germs in the nasal and buccal cavities. For this reason M. Servel experimented under different conditions. This time he operated on the liver and kidney of dogs which he killed by femoral hemorhage. After placing a ligature upon the hilus of the liver and kidney these organs were removed, their fibrous envelopes being respected throughout, and placed in a solution of chromic acid. After five days of immersion the surface of the organs was hardened, but the central portions were putrefied and filled with bacteria.

"These results," says the author, "prove two principal facts :"

"1. That the demonstration, by M. M. Bechamp and Estor, of the generation and evolution of bacteria in organic tissues protected from the air, is entirely correct."

"2. That the effect of conservative agents is the death of microzymas or surviving molecular elements of organs."

